# Valosin-Containing Protein (VCP)/p97: A Prognostic Biomarker and Therapeutic Target in Cancer †

**DOI:** 10.3390/ijms221810177

**Published:** 2021-09-21

**Authors:** Susan Costantini, Francesca Capone, Andrea Polo, Palmina Bagnara, Alfredo Budillon

**Affiliations:** Experimental Pharmacology Unit-Laboratory of Mercogliano (AV), Istituto Nazionale Tumori-IRCCS Fondazione G. Pascale, 80131 Naples, Italy; f.capone@istitutotumori.na.it (F.C.); a.polo@istitutotumori.na.it (A.P.); p.bagnara@studenti.unisa.it (P.B.)

**Keywords:** VCP, p97, AAA+ ATPase, cancer, prognostic biomarker, CB-5083

## Abstract

Valosin-containing protein (VCP)/p97, a member of the AAA+ ATPase family, is a molecular chaperone recruited to the endoplasmic reticulum (ER) membrane by binding to membrane adapters (nuclear protein localization protein 4 (NPL4), p47 and ubiquitin regulatory X (UBX) domain-containing protein 1 (UBXD1)), where it is involved in ER-associated protein degradation (ERAD). However, VCP/p97 interacts with many cofactors to participate in different cellular processes that are critical for cancer cell survival and aggressiveness. Indeed, VCP/p97 is reported to be overexpressed in many cancer types and is considered a potential cancer biomarker and therapeutic target. This review summarizes the role of VCP/p97 in different cancers and the advances in the discovery of small-molecule inhibitors with therapeutic potential, focusing on the challenges associated with cancer-related VCP mutations in the mechanisms of resistance to inhibitors.

## 1. Introduction

Valosin-containing protein (VCP/p97) was originally identified by Moir et al. (1982) in *Saccharomyces cerevisiae* and was strongly associated with cell cycle arrest; therefore, it has also been called Cdc48 (cell division cycle 48) [1]. In humans, VCP/p97 is one of the most highly expressed proteins and has been found in the brain, breast, heart, kidney, liver, lung, ovary, skeletal muscle, and testis. It is localized mainly in the endoplasmic reticulum (ER), belongs to the AAA+ ATPase family, and is known as the transitional endoplasmic reticulum ATPase (TER ATPase) [2].

**Figure 1:**
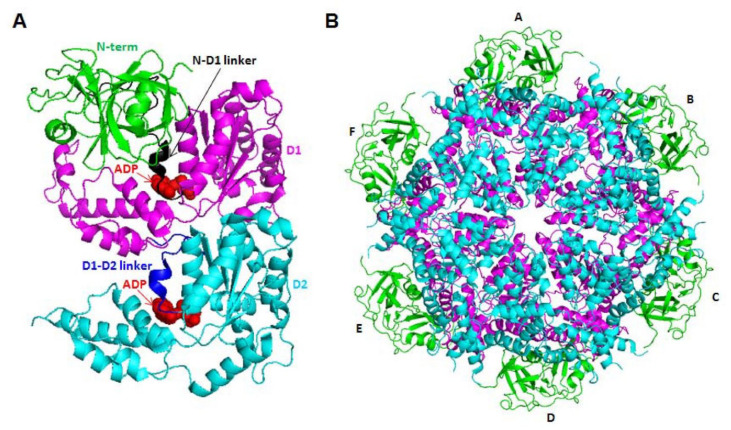
Molecular structures (PDB code: 5FTK chain A: 21–763 residues) of the VCP monomer (**A**) and hexamer (**B**). The N-terminal region is shown in green, N-D1 linker in black, D1 domain in magenta, D1-D2 linker in blue and D2 in cyan. ADP molecules are presented as red spheres.

The VCP/p97 structure comprises an N-terminal region (1–186 residues), two ATPase domains, D1 (209–460 residues) and D2 (481–763 residues), and a C-terminal region (764–806 residues). It functions as a homohexamer, and its active form is composed of a complex of a double ring structure, with the D1 and D2 domains sitting on top of each other (Figure 1) [3]. The N-terminal domain of VCP/p97 is involved in substrate recognition and interaction with cofactors, as described below, whereas the C-terminal domain is involved in nuclear localization. Regarding the two ATPase domains, D1 is implicated in oligomerization and the subsequent assembly of hexamers, whereas D2 catalyzes ATP hydrolysis [4]. One of the two linkers is located between the N-terminal region and D1, and the other is located between the D1 and D2 domains. Both linkers play a critical role in VCP/p97 function. The linker region between the D1 and D2 domains is essential for D2 ATPase activity [5], whereas that between the N-terminal region and D1, together with these two domains, is the location of most disease-associated VCP/p97 mutations [6,7].

**Figure 2:**
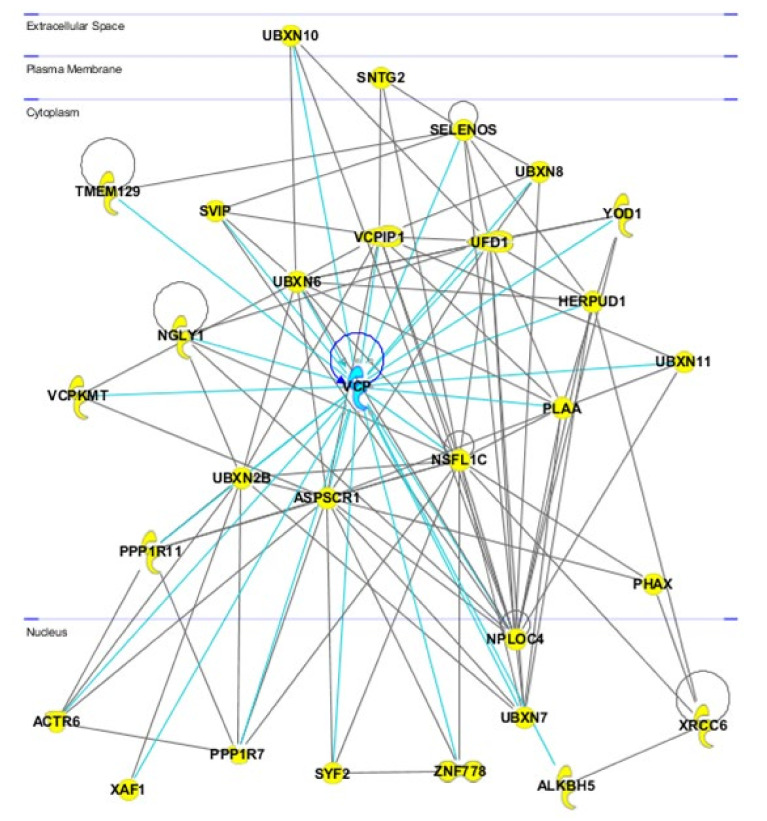
Protein−protein interaction network obtained using the Ingenuity Pathway tool showing the role of VCP as a hub node in cyan and all the correlated proteins in yellow symbols.

**Figure 3:**
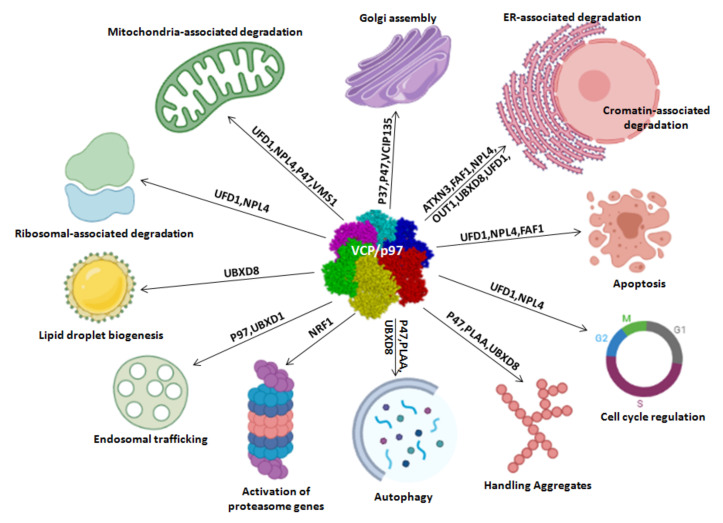
Biological functions of VCP/p97.

VCP/p97 is a multifunctional protein with a considerable effect on protein metabolism and intracellular homeostasis because it functions as a segregase to extract target proteins from the cellular organelle membrane, DNA complexes or protein complexes and promotes their degradation, refolding, recycling and relocation [8,9]. VCP/p97 interacts with multiple cofactors that are also directly correlated (Figure 2) and recruits proteins to different subcellular locations to participate in diverse cellular processes, such as endoplasmic reticulum-associated protein degradation (ERAD), chromatin-associated degradation, endosomal trafficking, aggregate trafficking, autophagy, induction of proteasome gene expression, lipid droplet biogenesis, ribosomal-associated degradation, mitochondria-associated degradation, apoptosis, cell cycle regulation and Golgi assembly (Figure 3) [10]. Multiple VCP/p97 cofactors share common binding motifs and conserved binding modules, such as the UBX (ubiquitin regulatory X) domain, UBXL (UBX-like) domain, VBM (VCP/p97-binding motif) and VIM (VCP/p97-interacting motif). These cofactors bind to different sites on VCP/p97 and guide the protein to different membrane structures to perform its biological functions [11]. For example, the cofactor alveolar soft part sarcoma locus (ASPL) relies on its extended UBX domain (eUBX) [12], whereas selenoprotein S (SELENOS) binds to the VIM domain and translocates VCP/p97 to the ER membrane during ERAD [13].

Among all the functions of VCP/p97, it associates with p47 or p37 to regulate Golgi and ER membrane fusion [14]. It also enables the removal of protein complexes from chromatin that are otherwise tightly bound to DNA, such as stalled RNA polymerase II [15,16] and the sterically trapped Ku70/80 complex [17].

VCP/p97 is a molecular chaperone involved in the ERAD pathway. It is recruited to the ER membrane by binding to membrane adapters such as nuclear protein localization protein 4 (NPL4), p47 and UBX domain-containing protein 1 (UBXD1) [18,19]. ATP binding to VCP/p97 induces a conformational change and subsequent unfolding of the target protein. Thus, VCP/p97 extracts misfolded proteins through its putative channel by ATP hydrolysis and subsequently delivers them to the proteasome for degradation [20,21], facilitating the translocation of target proteins into the narrow proteolytic core of the 26S proteasome [22]. The NPL4-UFD1-VCP complex is involved in cell cycle regulation and extraction of prereplication factors from chromatin in S phase and Aurora B in M phase [17,23].

Another role of VCP/p97 has been identified in the clearance of damaged lysosomes by autophagy. In fact, upon lysosome damage, VCP/p97 translocates to lysosomes and cooperates with phospholipase A2-activating protein (PLAA) and UBXD1 to promote autophagosome formation [24].

Recently, the ATPase activity of VCP/p97 was shown to enable the remodeling of topoisomerase 1 (TOP1) and facilitate the proteolytic cleavage of TOP1-DNA complexes by SPRTN metalloprotease [25].

## 2. Cellular Functions of VCP/p97

As reported in the previous paragraph and in Figure 3, VCP/p97 is involved in some cellular processes under physiological conditions, including protein quality control, cell cycle progression, cell death-related processes such as apoptosis and autophagy, chromatin-associated degradation, Golgi assembly, endosomal trafficking and lipid droplet biogenesis [26,27,28].

### 2.1. Protein Quality Control

VCP/p97 has been implicated in several pathways related to protein quality control, including ERAD, mitochondria-associated degradation, ribosome-associated degradation and proteasomal degradation.

The ERAD pathway eliminates misfolded proteins that are retrotranslocated into the cytosol through two essential steps [29]. In the first step, a portion of a substrate is moved across the lipid bilayer into the cytosol by a reaction mediated by a protein retrotranslocation complex containing the multispanning membrane ubiquitin ligase Hrd1 [30], whereas in the second step, VCP/p97 is recruited to the site of retrotranslocation via association with proteins such as SELENOS (VIMP), derlins and HRD1 [31]. Then, misfolded proteins undergo ubiquitination and are extracted from the membranes by VCP/p97 [32]. Eventually, these dislodged ERAD substrates are targeted for degradation by the proteasome [33].

Moreover, VCP/p97 facilitates mitochondria-associated degradation, a process that eliminates aberrant polypeptides from the mitochondrial outer membrane to maintain mitochondrial protein homeostasis [34]. In addition, upon mitochondrial damage, VCP/p97, UFD1, and NPL4 are recruited to the surface of mitochondria, as required for the clearance of damaged mitochondria by mitophagy [35]. A study identified a protein named VMS1 (VCP/p97-associated mitochondrial stress-responsive (1) that recruits VCP/p97 to mitochondria [36].

VCP/p97 is also involved in ribosome-associated degradation, a process that degrades aberrant nascent polypeptides stalled on ribosomes [37]. Importantly, ribosome stalling occurs due to a defect in mRNA translation caused by a lack of stop codons or truncation. VCP/p97 is recruited by a ribosome-associated factor named RQC1 together with the ubiquitinated substrate and extracts defective polypeptides from the ribosome to promote their degradation by the proteasome [38].

### 2.2. Cell Cycle

VCP/p97 was originally identified as a cell division cycle factor in yeast [1]. In mitosis, this protein plays roles in Aurora B kinase localization, DNA replication, microtubule spindle disassembly, chromosome segregation, and nuclear envelope reformation [39,40,41,42,43,44].

In detail, VCP/p97 associates with UFD1 and NPL4 to play a role in DNA replication initiation [39]. Depletions of VCP/p97 and UFD1 or NPL4 lead to replication stress and delayed cell cycle progression [39]. Based on these results, at least a couple of substrates for p97 likely exist in the cell division processes. VCP/p97 is required for cyclin E degradation [40], for the metaphase-to-anaphase transition by stabilizing separase, which cleaves cohesin components to separate sister chromatids [41], and for mitotic M phase [42].

VCP/p97 complexed with UFD1 and NPL4 binds to ubiquitinated Aurora B kinase and extracts it from chromatin, leading to chromatin decondensation and nuclear envelope reformation [43].

Sasagawa et al. found that VCP/p97 recognizes and extracts Aurora B kinase from chromatin during meiosis progression in *C. elegans* and guarantees the precise localization of Aurora B kinase to the cohesion sites of homologous chromatids at meiosis I prophase [44].

### 2.3. Cell Death: Apoptosis and Autophagy

VCP/p97 is essential for cell survival and proliferation and functions as an anti-apoptotic factor. In 1997, the expression of its variant (S565G mutation) in yeast decreased its ATPase activity and induced apoptotic cell death, indicating the involvement of this protein in apoptosis [45]. VCP/p97 depletion or mutations (at positions 251, 524 or 578) triggered apoptosis [46]. Based on these results, the mutations interfered with ERAD, induced the aberrant accumulation of polyubiquitinated proteins in the mitochondria, and impaired the degradation of dysfunctional mitochondria that in turn may also contribute to apoptosis by producing robust oxidative stress and triggering caspase activation. On the other hand, in 1999, VCP/p97 overexpression was reported to inhibit apoptosis in mammalian cells [47].

More recently, the function of VCP/p97 in the initiation of autophagy or maturation of autophagosomes has emerged. This protein interacts with LC3 and p62, the two most reliable biochemical markers of autophagy [48]. In 2009, researchers first reported the involvement of VCP/p97 in mammalian autophagy, particularly in patients with inclusion body myopathy associated with Paget disease of bone and frontotemporal dementia (IBMPFD), who had LC3-enriched vacuoles in muscle tissue [49]. The authors examined a mouse model expressing the mutated VCP/p97 gene and observed impaired autophagosome fusion with lysosomes because of p62 and LC3 accumulation [49]. In 2016, Yeo et al. showed that VCP/p97 inactivation in adult hippocampal neural stem (HCN) cells cultured in the presence of insulin impaired autophagosome maturation, whereas VCP/p97 depletion or inhibition in insulin-deprived cells with a high autophagy rate decreased autophagy initiation and activated apoptosis [50], suggesting different roles for VCP/p97 in early and late stages of autophagy. Moreover, Chung et al. reported that VCP mediates the crosstalk between autophagy and apoptosis based on calpain activity and intracellular Ca^2+^ levels [51].

### 2.4. Other Functions

VCP/p97 is also implicated in chromatin-associated degradation by linking nuclear substrates and releasing polypeptides from chromatin [52].

In mitotic cells, VCP/p97 regulates vesicle fusion at the exit of mitosis when the ER and Golgi apparatus may be reshaped [53] through the adaptors p37 [54], p47 [55] and p97-associated deubiquitinase (VCIP135) [56].

VCP/p97 regulates receptor-mediated endocytosis [57]. Some proteomic studies showed that VCP/p97 inhibition causes enlarged endosomes derived from increased early endosome-associated antigen 1 (EEA1) oligomerization, resulting in uncontrolled endosomal fusion [58]. Other authors reported that VCP/p97, together with UBXD1, interacts with caveolin, and endosomes were enlarged and the trafficking of caveolin to late endosomes was affected in VCP/p97-deficient cells [59].

Finally, VCP/p97 regulates the activity of adipose triglyceride lipase (ATGL), an enzyme that controls lipid droplet biogenesis [60].

## 3. VCP/p97 Expression and Function in Cancer

As summarized in Table 1, altered VCP/p97 expression has been identified in different cancer types and correlates with patient outcomes, as it is functionally associated with aggressiveness and therapeutic resistance (Table 1). Thus, VCP/p97 represents a potential prognostic biomarker and a therapeutic target.

### 3.1. VCP/p97 in Gastrointestinal Cancers

#### 3.1.1. Colorectal Cancer

In 2004, Yamamoto et al. analyzed patients with colorectal carcinoma using immunohistochemistry and found that tissues with hepatic metastatic foci expressed VCP/p97 at higher levels, whereas patients with colorectal adenoma expressed the protein at lower levels. Higher VCP/p97 levels correlated with a high invasion depth (T3–4), the presence of venous invasion and advanced tumor stage (III and IV). Moreover, patients with higher VCP/p97 expression also exhibited a significantly higher recurrence rate, whereas patients with lower expression showed better 5-year survival rates, with significant differences in disease-free survival rates between patients with stage II and III tumors [61]. Another group reported, in 2012, that serum VCP/p97 levels were elevated in patients with colon cancer. They compared VCP/p97 levels with those of other commonly used serum diagnostic markers, such as carcinoembryonic antigen (CEA), and revealed a strong correlation between CEA and VCP/p97 levels [62].

Moreover, VCP/p97 knockdown inhibits cell proliferation, chemoresistance and invasion and induces apoptosis in HCT116 colon cancer cells, whereas its overexpression suppresses apoptosis and chemoresponsiveness and promotes the proliferation and invasion of RKO colon cancer cells. Furthermore, VCP/p97 knockdown suppressedcarcinogenesis and metastasis “in vivo” in an HTC116 xenograft model through STAT3 dephosphorylation [63].

Very recently, the complex between VCP/p97 and ubiquitin-specific protease 11 (USP11) was verified to influence VCP/p97 expression and colon cancer-drug resistance in HTC116 colon cancer cells. In fact, USP11 promotes colorectal cancer cell resistance to chemotherapy (5-fluorouracil)by inducing VCP-dependent autophagy [64].

#### 3.1.2. Pancreatic Cancer

In the literature, few scientific investigations have reported a role for VCP/p97 in pancreatic cancer, one of most lethal malignancies. In 2004, Yamamoto et al. investigated the role of VCP/p97 expression in patients with pancreatic ductal adenocarcinoma (PDAC) undergoing curative resection. Using an immunohistochemistry approach, they showed that VCP/p97 expression correlated significantly with lymph node metastasis, which emphasized the role of this protein as an aggressiveness indicator in patients with PDAC. Moreover, these authors also highlighted that its expression levels were a prognostic marker for disease-free and overall survival in patients with early and advanced stages of PDAC [65].

Subsequently, the function of VCP/p97 has been analyzed in pancreatic cancer cells, xenograft cancer mouse models and patient cancer tissues, together with miR-198, which was found to directly and indirectly affect VCP/p97 expression. Specifically, Marin-Muller et al. (2013) showed that the downregulation of miR-198 in pancreatic cancer cells and tissue samples is associated with the overexpression of VCP and other tumorigenic factors (MSLN, OCT-2 and PBX-1). Hence, the increase in miR-198 levels in pancreatic cancer cells induces a decrease in tumor growth and metastasis and an increase in patient survival by directly targeting VCP and other factors. Therefore, these authors concluded that reduced miR-198 expression correlates with poor patient outcomes, whereas higher miR-198 levels predict a better prognosis and prolonged survival of patients with pancreatic cancer [66].

Because the increased storage of lipids in lipid droplets (LDs) is advantageous for the survival of cancer cells, Bai et al. showed in 2021 that LD-associated proteins correlate with the outcomes of patients with pancreatic cancer through a bioinformatics analysis. Specifically, they identified some differentially expressed LD-associated genes in patients with pancreatic cancer, including VCP. These findings underscore the importance of further studies examining the roles of LD-associated factors to develop more targeted therapies for patients with pancreatic cancer [117].

Among all pancreatic tumors, a small percentage are pancreatic endocrine neoplasms (PENs) originating from pancreatic islet cells and are characterized by the excessive production of endogenous hormones such as insulin or glucagon. VCP/p97 is also a prognostic factor for this cancer subtype. The utility of VCP/p97 and Ki-67 expression in predicting malignant behavior and the prognosis of patients with PENs and their correlations with clinicopathological characteristics have been reported [65].

#### 3.1.3. Liver Cancer

A correlation between elevated expression levels of VCP/p97 assessed using immunohistochemistry and shorter survival of patients with recurrent hepatocellular carcinoma (HCC) indicated that its expression levels had prognostic significance for determining the disease-free and overall survival of patients with HCC [67]. In 2012, another group was the first to describe that miR-129-5p suppresses VCP/p97 expression in HCC by regulating the NF-κB pathway. Specifically, VCP/p97 downregulation or miR-129-5p overexpression induces cell apoptosis, reduces cell growth and migration in vitro, and inhibits HCC development and progression in vivo [68]. A few years later, Fang et al. documented that long noncoding RNA nuclear-enriched abundant transcript 1 (NEAT1), which binds miR-129-5p, is overexpressed in HCC tissues and cell lines compared to normal cells and tissues, suggesting that it potentially represents a new target in HCC diagnosis and treatment [69]. Mechanistically, these authors revealed that the overexpression of NEAT1 induces miR-129-5p downregulation, VCP/p97 overexpression, and, hence, increased proliferation of HCC cells.

Moreover, Yi et al. (2012) examined liver cancer cells (HepG2 and HuH7) and showed that sorafenib, one of the first-line therapeutic options for patients with metastatic HCC, blocks tyrosine phosphorylation of VCP/p97, disrupts the cell secretory pathway and induces ER stress and autophagy-mediated cell death [70]. VCP/p97 is also a substrate of protein tyrosine phosphatase receptor type O (PTPRO) in HCC cells, based on a mass spectrometry analysis, and PTPRO suppression increases the phosphorylation of its substrate VCP/p97 and contributes to hepatocarcinogenesis [71].

In a recent paper, VCP/p97 was identified as a hub gene that plays an important role in the association between HCC and type 2 diabetes mellitus using bioinformatics approaches [118].

#### 3.1.4. Gastric and Esophageal Cancer

VCP expression was analyzed in patients with gastric cancer using immunohistochemistry, and 71.3% of the samples showing higher VCP expression were accompanied by a greater tumor size, the presence of vascular and lymphatic invasion and lymph node metastasis, and shorter overall and disease-free survival than those with lower VCP/p97 expression. The multivariate analysis also confirmed that VCP/p97 expression was a potential prognostic marker for overall and disease-free survival [72]. A proteomic analysis showed an increase in VCP levels in *Helicobacter pylori*-infected gastric adenocarcinoma cells (AGS) [73]. A few years later, a proteomics-based analysis identified eighteen VCP-interacting proteins in *H. pylori*-infected AGS cells. Therefore, infection increased the interaction between AKT and VCP/p97 and the phosphorylation of VCP/p97 promoted gastric epithelial cell survival, degradation of cellular regulators, and ultimately gastric carcinogenesis [74].

Moreover, Arai et al. (2016) showed a relationship between VCP/p97 and TRAIL resistance-overcoming activity in gastric adenocarcinoma (AGS) cells. These authors identified VCP/p97 as a target protein of 5′-I fuligocandin B, a natural product that overcomes TRAIL resistance. VCP/p97 knockdown in AGS cells induced TRAIL sensitivity, the overexpression of CHOP and DR5 proteins, and subsequent TRAIL-induced cell death [75].

Additionally, patients with esophageal squamous cell carcinoma (ESCC) present higher VCP/p97 expression, which is significantly correlated with higher frequencies of lymph node metastasis, deep invasion (pT_3_ and pT_4_), local recurrence and distant metastasis. In addition, patients with lower VCP/p97 expression have better 5-year survival rates and disease-free and overall survival than those presenting higher levels [76].

More recently, Luo et al.(2019) investigated the role of VCP/p97 in esophageal cancer treatment. In patients with locally advanced ESCC who were treated with radiotherapy, VCP/p97 expression was significantly increased in the cytoplasm of cancer cells when assessed using immunohistochemistry, and its elevated expression indicated shorter overall survival [77].

### 3.2. VCP/p97 in Breast Cancer

VCP/p97 also plays an important and crucial role in breast cancer, the most common cancer in women overall. In 2015, VCP/p97 overexpression was detected in breast cancer tissues compared to normal tissues using immunohistochemistry and correlated with decreased overall survival rates of these patients, which highlighted VCP/p97 as a useful prognostic biomarker in breast carcinoma [78].

Polyubiquitinated proteins are extracted from various cellular structures and compartments by VCP/p97 in an energy-dependent manner through the assistance of cofactors such as NPL4/UFD1, which facilitate their removal, recycling and degradation. In this context, Zhu et al. (2020) characterized a DNA damage-specific phosphorylation event of VCP (Ser784) mediated by members of the DNA damage response (DDR) kinase family that selectively improved VCP activity in chromatin-associated protein degradation, particularly its nuclear DDR functions. Mechanistically, these authors correlated the functional effects of Ser784 phosphorylation on the DDR, with a decrease in VCP/p97 phosphorylation linked to polyubiquitinated substrates, chromatin, and cofactors. They speculated that this phosphorylation might favor chromatin protein extraction and accelerate the separation of VCP/p97 from its partners to release substrates. From a clinical perspective, the authors associated nuclear pSer784-VCP levels with poor outcomes for patients with triple-negative breast cancer receiving chemotherapy but not with other types of therapy, suggesting that pSer784-VCP might be a potential predictive biomarker for the efficacy of chemotherapy [79]. Subsequently, the same authors maintained that low levels of pSer784-VCP should be considered a predictive biomarker for patients with breast cancer who are treated with genotoxic therapies and that patients with higher levels of pSer784-VCP might benefit from chemosensitization DDR kinase family inhibitors already in clinical use [80].

Recently, the role of VCP/p97 in an estrogen-positive breast cancer cell line (MCF7) has also been described. Its higher-than-normal expression in MCF7 cells inversely correlated with survival rates and regulated cell cycle progression from G0/G1 to S phase by degrading CDK inhibitors, including p21 and p27 [81].

Recently, Li et al. (2021) evaluated VCP/p97 expression in cancer stem cells (CSCs) from two human breast cancer cell lines (MCF7 and MDA-MB-231) and in human breast cancer tissues compared with non-CSCs. First, immunohistochemical staining of human breast cancer tissues showed higher expression of VCP/p97 than in normal tissues and its correlation with the histological grade, tumor size, and lymph node metastasis. Next, using flow cytometry and cell sorting, the cellular subsets CD44+/CD24−/low, aldehyde dehydrogenase (ALDH)+/high, and PKH26+ were identified to represent breast CSCs. VCP/p97 was expressed at higher levels in this cellular subset than in the respective non-CSC populations in human breast cancer tissues and cells, and VCP/p97 expression positively correlated with SOX2, a CSC marker. Using an orthotopic tumor model, breast cancer cells and mammospheres, the effects of VCP/p97 on cancer and CSC proliferation were examined. The authors described the mechanism of action of VCP/p97 in breast CSCs and suggested that its depletion or inhibition blocked the proliferation of ALDH+ CSCs or CSC-enriched mammospheres. The VCP/p97 deficiency activated the unfolded protein response and altered the expression of several stemness and pluripotency regulators, which together contributed to the disappearance of the CSCs [82].

### 3.3. VCP/p97 in Prostate Cancer

Over a decade ago, the role of VCP/p97 in patients with prostate cancer was examined, and higher VCP/p97 levels were associated with a poor prognosis. Specifically, VCP/p97 expression was evaluated using immunohistochemistry and transcriptome analyses in 136 patients with prostate cancer treated with conservative therapy, such as radiation, watchful waiting and androgen deprivation. These analyses showed a significant correlation between higher VCP/p97 expression and a poor prognosis and confirmed a correlation between VCP/p97 overexpression and an increased metastatic potential of tumor cells [83].

In the literature, many experimental and clinical data highlight an important role for interleukin 6 (IL-6) in prostate cancer development and progression. Serum IL-6 levels are increased in patients with metastatic or castration-resistant PCa (CRPC), positively regulate androgen receptor (AR) activity in a ligand-independent manner and induce the development of androgen-independent prostate cancer (AIPC). By performing a proteomic analysis of AR-independent prostate cancer cells (LNCaP) stimulated with IL-6 compared to normal cells, Duscharla et al. (2018) documented that VCP/p97 was overexpressed and that its IL-6-induced expression occurred through Pim-1 activation in LNCaP cells. These results suggested a role for VCP/p97 as a promising target in CRPC treatment. Interestingly, the overexpression of VCP/p97 by transfection in LNCaP cells promoted cellular proliferation, migration and invasion, whereas its inhibition induced the opposite effect and caused apoptosis through ER stress and cell cycle inhibition [84]. Recently, a novel VCP/p97-mediated survival mechanism based on the metabolic adaptation of cancer cells to starvation through the inhibition of mitochondrial activity was identified in the prostate cancer cell line PC3. In detail, under starvation conditions, VCP/p97 relocalizes and forms aggregate-like structures at perinuclear regions, mediating a reduction in mitochondrial activity and ROS production by the cells. This effect depends on glutamine depletion in the starvation medium because the readministration of glutamine restores the uniform distribution of VCP/p97 and triggers necrotic cell death through the ferroptotic pathway, which is associated with a decrease in GSH levels and a high intracellular ROS content [85].

### 3.4. VCP/p97 in Lung Cancer

VCP/p97 overexpression has also been detected in tissues from patients with non-small cell lung carcinoma (NSCLC). It correlates with the disease-free and overall survival of these patients and represents a prognostic marker for patients with both early and advanced stages of NSCLC [86]. Valle et al. (2011) were the first to identify a role for VCP/p97 in regulating key cellular processes involved in NSCLC initiation and progression. In particular, they confirmed higher expression levels of VCP/p97 in NSCLC tissues and cell lines compared to their normal counterparts, indicating that VCP/p97 was involved in NSCLC development, progression and metastasis through functional studies. Furthermore, its inhibition by si/shRNA or small-molecule inhibitors impeded the proliferation, migration and invasion of NSCLC cells, activated apoptosis by blocking the cell cycle in G0/G1 phase, and regulated TP53 through the proteasomal degradation pathway, all of which are likely mechanisms controlling tumor cell proliferation and progression [87].

In a pilot study, three single nucleotide polymorphisms (*rs1053318*, *rs2074549*, and *rs514492*) in the VCP/p97 gene were investigated to evaluate their effects on the clinical outcome of patients with advanced NSCLC treated with platinum-based chemotherapy. However, no significant correlation between the presence of VCP/p97 polymorphisms and treatment efficacy was found [119]. Other authors showed that the VCP/p97 gene is a target of miR-129 and is downregulated in human lung cancer, thus suggesting that miR-129 is a tumor suppressor miRNA that plays an essential role in the development and progression of this cancer. The overexpression of miR-129 induces a decrease in VCP/p97 levels in lung cancer cells (A549 and SPCA-1) [88].

Moreover, Shah et al. (2015) reported that siRNA-mediated VCP/p97 loss activated ER stress and induced the epithelial-mesenchymal transition (EMT) and apoptosis in lung adenocarcinoma cell lines (A549, H358 and HPLD-1). In particular, these authors documented decreased levels of the VCP protein in nearly all primary lung tumors and increased levels of ER stress and EMT markers, suggesting the clinical potential of these observations, because increased levels of ER stress and EMT markers contribute to chemoresistance, and hence correlate with the shorter survival of patients [89].

### 3.5. VCP/p97 in Bone Cancers

The first paper related to the evaluation of VCP/p97 expression in osteosarcoma was published in 2002. The authors reported its overexpression in murine osteosarcoma cells with higher metastatic potential (LM8), but not in parental cells that did not form pulmonary metastasis, and its involvement in the regulation of the NF-κB signaling pathway [90]. VCP/p97 is downregulated in liver-bone-kidney alkaline phosphatase (L/B/K ALP) and CD99 glycoprotein-transfected osteosarcoma cells, which show a low metastatic ability, and this observation correlates with decreased NF-κB activity [91]. Moreover, He et al. (2015) identified a positive correlation between the protein levels of phosphorylated VCP and Aurora-B in osteosarcoma tissues using immunohistochemistry; Aurora-B knockdown suppresses the phosphorylation of VCP, NF-κB activity, and the malignant phenotype of osteosarcoma cells (U2-OS and HOS) [92]. In parallel, Long et al. (2015) found a significant negative correlation between lower levels of miR-129-5p and higher levels of VCP/p97, a target of miR-129-5p, in osteosarcoma tissues with pulmonary metastasis. Thus, approaches targeting the VCP/miR-129-5p signaling pathway might represent a therapeutic strategy for osteosarcoma management [93]. Moreover, the authors of a recent study modulated VCP/p97 expression and observed a connection with autophagy induction, anoikis resistance and osteosarcoma cell metastasis [94]. An interesting study assessing Saos-2 osteosarcoma cells highlighted a novel regulatory mechanism mediated by the serum amyloid p (SAP) protein, an apoptotic inducer that binds to VCP/p97, precluding it from exerting an anti-apoptotic effect [95]. VCP/p97 is also a substrate of protein tyrosine phosphatase L1 (PTPL1), and tyrosine phosphorylation promotes the tumorigenesis of Ewing sarcoma cells [96].

### 3.6. VCP/p97 in Head and Neck Cancers

VCP/p97 expression has also been examined in gingival squamous cell carcinoma (GSCC) tissues using immunohistochemistry. Strong staining was observed in 67.6% of cases and correlated positively with tumor stages. A multivariate analysis revealed that VCP/p97 levels, lymph node metastasis, and pT (TNM) were independent factors predicting overall survival [97]. The overexpression of VCP/p97 has also been detected in oral cancer, and its knockdown reduces cell proliferation and growth rates in oral cancer cells (SCC-9). In parallel, another oral cancer cell line (Cal-27) overexpressing VCP/p97 presented a significant increase in cell proliferation, the number of colonies formed in soft agar and colony size. Moreover, VCP/p97 is upregulated in carcinoma in situ lesions and invasive cancer tissues of oral squamous cell carcinoma (OSCC) compared to matched normal tissues [98].

VCP/p97 expression has also been determined in patients with oropharyngeal squamous cell carcinoma (OSCC) using immunohistochemistry, and its putative correlation with HPV-DNA has been analyzed. Although no correlation was detected between the HPV status and VCP/p97 expression, the authors reported that VCP/p97 overexpression in HPV-negative patients was associated with a significantly better 5-year disease-free survival rate [99].

Guo et al. (2017) found that VCP/p97 polymorphisms (rs2074549) affected the clinical outcomes of chemoradiotherapy in Chinese patients with nasopharyngeal carcinoma because they were significantly associated with the occurrence of myelosuppression. Hence, the authors suggested that the polymorphisms predict the clinical outcomes of chemoradiotherapy in Chinese patients [100].

### 3.7. VCP/p97 in Thyroid Cancer

VCP/p97 expression has also been evaluated in patients with follicular or papillary thyroid carcinoma compared to noncancerous tissues using immunohistochemistry. VCP overexpression significantly correlates with histological subtypes. Specifically, in patients with follicular thyroid carcinoma, VCP/p97 expression correlates with the pTclassification, lymph node metastasis and extrathyroidal extension. Patients with early-stage tumors show lower VCP/p97 expression. The prolonged overall and disease-free survival of patients with a low expression of VCP/p97 compared to patients with intermediate and higher VCP/p97 expression indicates that its overexpression was a prognostic marker for disease recurrence in patients with follicular thyroid carcinoma [101]. In 2018, other authors exposed female mice to bisphenol A (BPA) in utero and early in postnatal life and identified BPA-responsive proteins in thyroid tissues by performing proteomics analysis to study the risks of environmental exposure to this endocrine-disrupting chemical and its correlation with an increased thyroid cancer risk in women. Their results revealed a group of overexpressed proteins, including VCP, in murine thyroid tissues after BPA exposure. Moreover, the authors also showed a significant correlation between BPA levels in the blood of patients with thyroid cancer and VCP/p97 expression in thyroid cancer tissues, although the expression levels of the VCP/p97 mRNA in thyroid cancer were not significantly different from those in normal tissues. Therefore, they suggested that BPA might induce cancer progression rather than initiation and that VCP-mediated inflammatory pathways might disrupt thyroid function [102]. Recently, VCP/p97 was shown to govern the proteolysis of sodium iodide symporter (NIS), which is necessary for iodide uptake and critical for the efficacy of ablative radioiodine (RAI) treatment of thyroid cancer. Notably, using VCP inhibitors, VCP-mediated repression of NIS function was abrogated and subsequently resulted in markedly increased RAI uptake in mouse and human thyroid models, suggesting a new route to enhance the effectiveness of RAI therapy. Furthermore, a detailed analysis of the VCP/p97 expression profile and clinical relevance in thyroid cancer, using the TCGA papillary thyroid cancer (PTC) dataset, revealed that (i) VCP/p97 was significantly elevated in patients with PTC, particularly patients with RET (rearranged during transfection) fusion, BRAF-mutant, or PAX8 (paired box gene 8) fusion; and (ii) higher levels of VCP/p97 expression correlated significantly with shorter disease-free survival and an increased risk of recurrence in individuals with PTC subjected to ablative RAI treatment [103].

### 3.8. VCP/p97 in Hematological Cancers

High VCP/p97 expression correlates with shorter overall and disease-free survival of patients with orbital B-cell lymphomas. Moreover, VCP/p97 downregulation by an siRNA induces apoptosis and suppresses B-cell lymphoma cell invasion [104]. Additionally, in multiple myeloma (MM), VCP/p97 has been considered a potential therapeutic target, and numerous studies have reported the potential of small-molecule VCP/p97 inhibitors [105]. Furthermore, higher levels of VCP/p97 were identified in poor vs. good prednisone responders by analyzing leukemic blasts in patients with childhood acute lymphoblastic leukemia (ALL) [106]. Similarly, another study confirmed a role for VCP/p97 in glucocorticoid-resistant leukemic cells [120]. Notably, a proteomic approach indicated that VCP/p97 was the most abundant protein involved in exosome generation and secretion in T-cell leukemia Jurkat cells; indeed, VCP/p97 inhibition prevented tumor exosome secretion, thus highlighting a potential novel mechanism of VCP/p97 in tumorigenesis [107].

### 3.9. VCP/p97 in Other Cancers

Only two studies have documented the involvement of VCP/p97 in melanoma models. In the first study, a proteomic analysis revealed the overexpression of VCP/p97 upon irradiation of the human metastatic melanoma cell line BLM, which was potentially associated with a survival mechanism [108]. In contrast, in the second study using a coculture model of melanoma cells expressing Melan-A/MART-1 tumor antigen with Melan-A/MART-1_26-35_-specific cytotoxic T lymphocytes (CTLs), deregulation of VCP/p97 was associated with the immune escape mechanism. Indeed, VCP/p97 re-expression in resistant melanoma cells completely restored immune recognition by Melan-A/MART-1_26-35_ CTLs [109].

In 2013, some authors investigated the association of VCP/p97 with DNA-dependent protein kinases (DNA-PKs) and the effect of VCP/p97 regulation on the radiosensitivity of glioblastoma cells. VCP/p97 was phosphorylated by DNA-PKcs and its knockdown resulted in the accumulation of DNA-PKs in glioblastoma cells, increased DNA-PK activity, and a reduced survival time of xenografted mice with radiation treatment compared to the control group [110]. A similar result was reported by Biau et al. in 2017. Using a reverse-phase protein array approach, VCP/p97 was identified as one of several biomarkers of radioresponse in glioma subcutaneous xenografts and in vivo resistance to radiotherapy [111]. In the same year, other authors observed that the balance of HDAC6-VCP/p97 (increasedHDAC6levels and decreased VCP/p97 levels) was positively associated with temozolomide resistance in glioblastoma and that the reversal of the ratio of HDAC6-VCP/p97 represented a potential therapeutic strategy [112].

In 2016–2017, some studies reported VCP/p97 and UPS as targets of vulnerability in ovarian cancer. Specifically, VCP/p97 was identified as an essential gene in ovarian cancer cells and in cyclin E1-overexpressing cisplatin-resistant ovarian cancer cells [121,122]. In a recent paper, VCP correlated with the chemotherapy response in patients with high-grade serous ovarian carcinoma (HGSOC) receiving the platinum-taxane combination [113]. Moreover, through mass spectrometry-based proteomic profiling, Laguë et al. (2012) identified differentially expressed proteins in human ovarian granulosa cell tumors (GCTs) in a transgenic mouse model. They chose this approach because few tissue samples were available for this tumor, which represents only 5% of all ovarian cancers. VCP/p97 was among the most abundant secreted proteins in both cultured mouse GCT cells and in the serum of patients with GCTs compared to healthy controls. Therefore, these authors suggested that serum levels of VCP/p97 are a potentially useful marker for various clinical cancer applications [62].

Little is known about the role of the VCP/p97 protein in testicular cancer. Recently, Nakkas et al. investigated the expression of VCP/p97 in human testicular tumors and cancer-adjacent normal testicular tissues using immunohistochemistry. They confirmed higher VCP/p97 expression in different types of human testicular tumors, including intratubular and testicular germ cell tumors, yolk sac tumors, embryonal carcinoma, Sertoli cell tumors and Leydig cell tumors, and a negative correlation between VCP/p97 and autophagy markers (LC3B and p62) [114].

Kilgas et al. (2021) evaluated the role of VCP/p97 in bladder cancer cell lines and xenograft models. Significantly higher VCP/p97 expression was detected in muscle-invasive bladder cancer than in both normal tissue and papillary bladder cancer samples. Higher levels of VCP/p97 expression in patients with bladder cancer correlated with shorter survival following bladder removal by cystectomy. Moreover, VCP/p97 interacted with the MRE11-RAD50-NBS1 (MRN) complex on chromatin and was involved in its inactivation by blocking the disassembly of the MRN complex at the sites of DNA damage upon exposure to ionizing radiation [115].

McHugh et al. (2020) evaluated the effect of VCP/p97 on the viability of three squamous cell carcinoma (cSCC)cell lines (SCCRDEB4, SCCRDEBMet, and SCCTMet) compared to two normal skin cell lines (NHF and NHK) and reported that VCPsiRNAs killed cSCC cells but not normal skin cells. Cell death caused by VCP depletion was attenuated by the suppression of proteins involved in the response to the accumulation of unfolded proteins in the ER (ATF6, IRE1a/JNK1, and PKR/eiF2α) and amino acid depletion (GCN2/eiF2α) [116].

## 4. Inhibitors of VCP

Since VCP has important molecular and cellular roles and is involved in diverse physiological and pathological conditions, such as cancer, it is an interesting and potential therapeutic target. Therefore, many researchers are attempting to design or discover molecules that inhibit VCP, including quinazolines, pyrimidines, triazoles/thiazoles, indole derivatives, CB5083, and eeyarestatin I [123]. The next few paragraphs will provide an overview of the currently known inhibitors targeting VCP.

### 4.1. Quinazolines

Quinazolines are heterocyclic organic compounds containing benzene rings fused to pyrimidine nuclei and are classified into quinazolin-2(1H)-one and quinazolin-4(3H)-one compounds based on the position of the keto- or oxo-group. These molecules belong to the most prominent class of compounds that possess a broad range of pharmacological activities, such as analgesic, antioxidant, anti-inflammatory and anticancer activities [124]. Five known VCP inhibitors are classified into this category: N2,N4-dibenzylquinazoline-2,4-diamine (DBeQ), ML240, ML241, FQ393 and OSSL_325096.

In 2011, Chou et al. conducted high-throughput screening (HTS) to identify VCP inhibitors using a library of compounds from the Molecular Libraries Small Molecule Repository at the National Institutes of Health. From these analyses, DBeQ was the first selective and reversible VCP inhibitor able to block proteasomal degradation of VCP-dependent UPS substrate and autophagic degradation of LC3-II and to activate autophagic/apoptotic cell death [125,126].

In 2012, Yi et al. treated two HCC cell lines (HepG2 and HuH7) with DBeQ and sorafenib, a kinase inhibitor used for HCC treatment, and documented the synergistic effect of these two molecules on apoptosis activation and LC3-II accumulation [70]. Moreover, in 2014, Nashimura et al. reported that DBeQ cooperated with bortezomib to induce apoptosis in myeloma cells [127]. This molecule was also capable of facilitating the degradation of NF-κB subunit p100 (NFKB2) in HEK293T cells and decreasing proliferation in Raji lymphoma cells by downregulating the transcription of NFKB2 [128]. Experimental data from NSCLC cells (H1299) revealed that DBeQ decreases cell proliferation and migration but increases apoptosis compared to untreated control cells. Moreover, these authors confirmed their results using dendrimer-encapsulated DBeQ [129]. Recent studies showed that DBeQ induced LC3-II and p62 accumulation in the cytosol of mouse Sertoli cells, which are nurse cells of the testicles, and induced cell vacuolation, promoting cell death, apoptosis and cell cycle arrest [130]. Moreover, treatment of breast cancer cells (MCF7) with DBeQ arrests the cell cycle in G1 phase, followed by a blockade of p21 and p27 degradation [81]. Finally, in 2021, Desdicioglu R. et al. treated a human placental choriocarcinoma cell line (Jeg3) with DBeQ and observed the accumulation of the autophagic proteins LC3II and p62 and the inhibition of cancer cell growth through its effects on caspases and arresting the cell cycle [131].

In addition, Chou et al. (2013) used the previously identified quinazoline scaffold to explore the structure−activity relationships (SARs) of its different moieties and developed two new inhibitors, ML240 and ML241. Although both inhibitors exerted similar effects on the ERAD pathway, experimental studies revealed that only ML240 inhibited the autophagic degradation pathway by promoting LC3-II accumulation and caspase activation to induce rapid cell death, whereas ML241 did not show this activity [132]. In 2015, the same authors conducted a screen of many VCP/p97 inhibitor analogs to evaluate their ability to inhibit the ATPase activity of D1 and D2 domains. P47 served as the main cofactor of VCP/p97, and the p47-VCP complex affected the potency of ML240 and ML241, which highlighted the possibility of developing domain-selective and complex-specific VCP/p97 inhibitors [133].

Interestingly, T-cell leukemia Jurkat cells are more sensitive to cell death induced by DBeQ than normal T cells [107].

More recently, a new VCP/p97 ATPase activity inhibitor, OSSL_325096, with a chemical structure similar to DBeQ, was identified using a cell-based screen designed to identify novel small molecules against multiple myeloma (MM). Notably, this compound showed antiproliferative and apoptotic activity in MM cells, including bortezomib-resistant cell lines and primary myeloma cells, and blocked the growth of subcutaneous myeloma cell tumors in vivo [105].

Finally, the novel VCP/p97 inhibitor FQ393 was recently synthesized based on theDBeQ structure by replacing benzylamine with tryptamine. FQ393 was capable of inhibiting cell proliferation and inducing apoptosis in many cancer cell lines, including breast carcinoma (MCF7 and MDA-MB-231), cervical carcinoma (HeLa), colorectal carcinoma (DLD-1, HCT-8 and HCT-116), hepatoma (HepG2), lung adenocarcinoma (95D, A549, H1299, H1792, H1975, and H460), myeloma (MM1S), pancreatic carcinoma (BxPC-3 and PNAC-1), and skin cancer (A431) [134].

### 4.2. CB-5083

In 2015, CB-5083 was identified as a potent VCP/p97 inhibitor through extensive chemical optimization using the DBeQ and ML240 scaffolds as starting points. In vivo and in vitro studies showed that it bound to the D2 domain, inhibited VCP with moderate oral bioavailability, rapidly induced the UPR, induced apoptosis and inhibited tumor growth in hematological xenograft models [135,136]. Studies conducted using MM cell lines and patient-derived MM cells revealed that CB-5083 induced a significant decrease in viability in the majority of cell lines and not only inhibited tumor growth alone but also synergized with other drugs commonly used to treat patients with MM [137]. Furthermore, Gugliotta et al. reported the significant inhibition of the growth of ten B acute lymphoblastic leukemia cell lines by CB-5083; in particular, it was able to reduce colony formation by two cell lines (OP1 and NALM6) and GRP78−/−, GRP94−/−, and XBP1−/− cells. Moreover, CB-5083 induced ER stress by activating chaperones, increasing the activation of IRE1-alpha and PERK, and inducing the overexpression of CHOP and its downstream genes [138]. Studies of mantle lymphoma cells showed that treatment with CB-5083 combined with an HDAC6 inhibitor (ACY-1215) induces CDK4 and cyclin D1 downregulation, DNA damage and apoptosis. Furthermore, cotreatment with CB-5083 and ACY-1215 inhibitors reduces tumor volumes and prolongs the survival of NSG models with Z138C and Jeko-1 xenografts [139]. Recently, most osteosarcoma cell lines were reported to express VCP/p97 at higher levels than normal cells and that CB-5083 treatment leads to cytotoxicity in all osteosarcoma cell lines and cell cycle arrest with the inhibition of proliferation and colony formation [140]. In addition, Her et al. identified thrombospondin-1 (THBS1) as a CB-5083-upregulated gene involved in HCT116 colon cancer cell resistance to CB-5083, and THBS1 blockade sensitized cells to CB-5083-mediated growth inhibition and induced an increase in a sub-G1 population and caspase 3/7 activity [141]. Very recently, CB-5083 has also been tested in pancreatic cancer, which is known to carry TP53 mutations, among which the most frequent is Arg273His. Specifically, Wang et al. reported that inhibition of VCP/p97 by CB-5083 increases the ubiquitination and degradation of p53-R273H and induces cell death [142].

Moreover, Kilgas et al. showed that inhibition of VCP/p97 by CB-5083 increased bladder cancer cell killing after exposure to ionizing radiation and suppressed xenograft tumor growth without additional toxicity, except for acute intestinal toxicity due to ionizing radiation [115]. In 2021, a second-generation CB-5083 inhibitor, CB-5339, was developed and validated in multiple AML models, such as syngeneic and patient-derived xenograft murine models. The combination of CB-5339 with a DNA-damaging agent exerted a synergistic effect on an MLL-AF9-driven AML murine model [143].

CB-5083 was the first selective VCP inhibitor that showed promising preclinical activities, and two phase I clinical trials in advanced solid tumors (NCT02243917) and lymphoid hematological malignancies (NCT02223598) have concluded. However, these trials were discontinued and terminated due to adverse effects on vision resulting from inhibition of phosphodiesterase-6 (PDE6). Nevertheless, chronic administration of CB-5083 did not cause permanent retinal damage, which suggested a decrease in CB-5083 doses, and the re-evaluation of this compound as a clinical agent might be appropriate [144].

Currently, the second-generation CB-5339 compound has entered two phase I clinical trials for locally advanced or metastatic malignant solid tumors and aggressive or indolent non-Hodgkin lymphomas (NCT04372641) and acute myeloid leukemia or myelodysplastic syndrome (NCT04402541).

### 4.3. Pyrimidines

Pyrimidines are heterocyclic organic compounds and essential constituents of all cells. In medical chemistry, these molecules represent some of the most important compounds due to their remarkable pharmacological activities, such as anticancer, antiviral and antioxidant activities [145,146]. Pyrimidines have been developed as VCP/p97 inhibitors, including 2-alkylsulfanylpyrimidine. In 2014, Cervi et al. evaluated the SAR of a new class of reversible VCP inhibitors starting from the chemical modification of 2-alkylsulfanylpyrimidine. Some of these derivatives were tested on colon cancer cell lines (HTC-116) and showed antiproliferative activity [147]. In 2019, Wang et al. designed, synthesized and evaluated a new series of VCP/p97 pyrimidine inhibitors and identified that the compound N-(1-(4-(benzylamino)-7,8-dihydro-5H-pyrano[4,3-d]pyrimidin-2-yl)-2-methyl −1H-indol-4-yl)methanesulfonamide exerted a good antiproliferative effect on non-small cell lung cancer cells (A459) and showed good liver stability in mice, dogs and humans [148]. Very recently, new VCP pyrimidine inhibitors were designed, synthesized and biologically evaluated, and the compound (3-(((2-(2-methyl-4-(methylsulfonamido)-1H-indol-1-yl)-6,7-dihydro-5H-cyclopenta[d]pyrimidin-4-yl)amino)methyl)phenyl)boronic acid) showed good enzymatic activity [149]. In particular, investigations using non-small cell lung cancer (A549) and multiple myeloma (RPMI8226) cells revealed its potential effect on the inhibition of cellular proliferation in both cell lines [150]. Finally, other authors developed and designed other covalent inhibitors that target the D2 domain of VCP/p97 starting from pyrazolo[3,4-d]pyrimidine (PP). Among them, the compound (N-(1-(tert-butyl)-3-phenyl-1H-pyrazolo[3,4-d]pyrimidin-4-yl)acrylamide, named PPA, showed potent inhibition of VCP/p97. Moreover, analyses of two mutants of Cys522 (Cys522Ala and Cys522Thr) located in the D2 domain, which is the most important target of multiple VCP inhibitors, during PPA inhibition showed that PPA blocked cell proliferation. In addition, these authors reported that PPA exerted antiproliferative effects on colon cancer (HCT116), cervical cancer (HeLa), and multiple myeloma (RPMI8226) cells and inhibited the growth of HCT116 cells resistant to two other inhibitors (CB-5083 and NMS-873), suggesting that PPA is a good candidate to treat cancer and overcome resistance to other VCP/p97 inhibitors [151].

### 4.4. Triazoles and Thiazoles

Triazoles and thiazoles are heterocyclic organic compounds with diverse biological activities, such as anti-inflammatory, antifungal, antiviral, and anticancer activities [152]. Specifically, thiazoles are unique heterocyclic compounds containing both sulfur and nitrogen atoms in their structures [149,153,154]. The two triazole and thiazole categories have been studied for their capacity to inhibit VCP/p97. In 2010, the compound 2-anilino-4-aryl-1,3-thiazoles was discovered through an HTS assay by Bursavich et al. and described for the first time as a potent VCP/p97 inhibitor with submicromolar activity against VCP [155]. On the other hand, in 2013, Polucci et al. described a series of alkylsulfanyl-1,2,4-triazoles capable of inhibiting the ATP activity of VCP/p97 and blocking the proliferation of HCT-116 cells. In particular, 3-[3-cyclopentylsulfanyl-5-(4′-methanesulfonyl-2-methyl-biphenyl-4-yloxymethyl)-[1,2,4]triazol-4-yl]-pyridine was indicated as the most promising compound [156].

One of the most frequently studied VCP/p97 inhibitors falling into the thiazole category is NMS-873. It was designed and identified in 2013 by Magnanghi et al. through an HTS assay using a one-million-compound library. The compound was capable of inducing a dose-dependent accumulation of poly-Ub proteins, stabilizing cyclin E and Mcl-1, and blocking the proliferation of multiple cancer cell lines (HeLa, U2OS and HCT116) [157].

### 4.5. Indole Derivatives

Another class of interest in medicinal chemistry is heterocyclic compounds derived from indole cores with interesting pharmacological properties, such as antiviral, antituberculosis, anticonvulsant and anticancer properties [158]. These molecules are also capable of inhibiting NF-κB/mTOR/PI3K/AKT, regulating estrogen-mediated activity and exhibiting antineoplastic properties [159]. Some inhibitors in the indole category were found to inhibit VCP/p97.

In 2016, Alverez et al. identified the indole compound SMDC818909 by HTS screening as a promising VCP inhibitor with excellent pharmaceutical properties, high solubility, permeability, and human microsomal stability. HTS assays on the NCI-60 panel of cancer cell lines showed an effect on multiple myeloma (RPMI-8226), breast cancer (MDA-MB-468) and melanoma (LOX IMVI) cells [160]. Another study focused on pentafluorosulfanyl (SF_5_) and trifluoromethyl indole (CF_3_) compounds and their derivatives and showed that pentafluorosulfanyl- and nitro-derivatives exhibited a 430-fold difference in VCP/p97 inhibitory activities [161]. In 2018, the same group conducted chemistry studies to optimize the side chain of the phenyl indole core using HTS and reported that the addition of an N-alkyl piperazine to the side chain of 2-phenylindole conferred high potency to this series of compounds. SAR analyses coupled with molecular modeling were used to develop other analogs that exerted antiproliferative effects on the NCI-60 panel of cancer cell lines (leukemia, NSCL cancer, colon cancer, CNS tumor, melanoma, ovarian cancer, renal cancer, prostate cancer, and breast cancer) using nanomolar to micromolar concentrations [162]. One of the most promising compounds identified was UPCDC-30245, an allosteric VCP/p97 inhibitor that binds to the interface of the D1 and D2 domains of VCP/p97 and prevents the conformational changes in VCP required for its function [163].

### 4.6. Eeyarestatin I

Eeyarestatin I (EerI) was first discovered in 2004 by Fieger et al. [164]. It contains two functional domains, one of which is an aromatic domain with ER membrane-binding function and the other is a nitrofuran domain required to bind the D1 domain of VCP/p97 [165]. This compound affects ERAD by interfering with deubiquitylating enzymes [166] and inhibits Sec61-mediated protein translocation and N-glycosylation of the P2X2 purinergic receptor in vitro [167]. Furthermore, the 5-nitrofuran (5-NF) ring of EerI impairs calcium homeostasis by increasing Ca^2+^ leakage from the ER [168]. In cancer, Wang et al. documented that the combination of EerI and bortezomib induces ER stress-mediated apoptosis in human B-cell lymphoma (HBL-2) and mantle cell lymphoma (JeKo-1) by promoting NOXA expression and inhibiting H2A ubiquitylation [169] and by inducing the overexpression of the proapoptotic protein CHOP in cervical cancer cells [127,170]. Moreover, Wang et al. showed that EerI collaborates with M1 oncolytic virus to induce HCC cell death by promoting ER stress-induced apoptosis and suppressing the inositol-requiring enzyme 1a (IRE1a)– X-box binding protein 1 (XBP1) pathway [171].

Very recently, Du et al. investigated whether EerI synergized with farnesyl thiosalicylic acid (FTS), an RAS inhibitor used to treat PDAC. The combination of EerI and FTS upregulated marker genes of the UPR and induced the apoptosis of murine and human PDAC cells [172].

### 4.7. Other Known Inhibitors

Limited published information is available for several additional VCP/p97 inhibitors, such as xanthohumol, MSC1094308, NW1028, NW1030, ebastine, astemizole, clotrimazole and NPD8733.

Xanthohumol (XN) is a prenylated chalcone present in beer and *Humulus lupulus* L. with the ability to inhibit VCP/p97 by binding the N domain, thus impairing the function of autophagosomes and the accumulation of microtubule-associated protein 1 LC3-II [173]. Subsequently, the antitumor activity of XN was confirmed in different cancer cell lines (A431, EC17, PC-3, EC109, HeLa, HEK293T, LoVo, HT29, Colo-201, HCT116, LS-174T, SW620, DLD-1, SW48, SW480, A549, MCF7 and A2058) [174].

In 2019, two other small molecules (NW1028 and NW1030) targeting the N-terminus and the D1 domains of VCP/p97 were identified using PNA-encoded chemical libraries. NW1028 inhibited the degradation of a VCP-dependent reporter, whereas NW1030 increased its degradation. However, treatment with NW1028 and NW1030 affected the mitotic process in HeLa cells, thus revealing a role for VCP/p97 in the regulation of mitotic spindle orientation [175].

In 2017, Segura-Cabrera et al. repurposed existing drugs using a structure-based virtual screening and in vitro assay and revealed that ebastine, astemizole and clotrimazole alter the ATPase activity of VCP/p97; specifically, clotrimazole inhibited the catalytic activities of both D1 and D2 domains, while ebastine inhibited only the activity of the D1 domain and astemizole inhibited only the activity of the D2 domain [176]. Regarding these three compounds, ebastine sensitizes NSCLC cells to chemotherapy and reverses drug resistance in NSCLC, breast and prostate cancer cells [177], whereas astemizole exhibits antiproliferative activity in melanoma and cervical, hepatocellular, lung, and breast cancers [178,179].

Finally, in 2019, a screen of ∼16,000 small compounds from the RIKEN NPDepo chemical library identified the NPD8733 compound as capable of binding to the D1 domain of VCP/p97 [180].

## 5. VCP/p97 Mutations and Their Involvement in the Mechanism of Inhibitor Resistance

Another interesting point of discussion on VCP/p97 pertains to the presence of mutations. A detailed analysis of the somatic mutations in VCP/p97 in human cancers, performed by the Catalog of Somatic Mutations in Cancer (COSMIC) [181], documented the presence of deletions (frameshift and in-frame), insertions (frameshift) and substitutions (coding silent, missense and nonsense mutations) located mainly in the N-terminal, D1 and D2 regions (Table 2).

The mechanism of resistance to VCP inhibitors in cancer has been reported to be linked to VCP/p97 mutations. Bastola et al. established CB-5083-resistant ovarian cancer cells that showed five- to six-fold higher resistance in vitro compared with parental cells. They analyzed genomic and complementary DNA sequences of the VCP/p97 coding region and found a pattern of coselected mutations. In one copy, missense mutations located at codon 470 in the D1-D2 linker increased ATPase activity, whereas in the other copy, nonsense or frameshift mutations located at codon 606 or codon 616 in the D2 domain caused the loss of allele-specific expression. These authors indicated that CB-5083 was approximately 3.4- to 3.8-fold less active in inhibiting ovarian cells with missense mutations at codon 470 compared to cells expressing wild-type VCP/p97 [121]. In 2019, Bastola et al. performed a global transcriptomic analysis of the parental and previously mentioned CB-5083-resistant cells and found additional mutations in the resistant cells. They detected two missense mutations located in the D1−D2 linker (Glu470Lys and Glu470Asp) in a resistant ovarian cell line (OVSAHO) but not in parental cells [182]. In addition, they also identified 71 unique nonsynonymous substitutions in resistant cells. Since CB-5083-resistant OVSAHO cells harbored additional mutations, these authors performed CRISPR-Cas9-based gene editing of VCP/p97 to produce both Glu470Lys and Glu470Asp mutants in HEK-293T cells, showing that these mutations increased resistance to CB-5083 by at least 12-fold compared to parental HEK-293T cells. Furthermore, colon cancer cells (HCT116) presented a heterozygous frameshift mutation at codon 616 (N616fs*), and HCT116-resistant cells were approximately 34 times more resistant to CB-5083 than parental cells [182].

Moreover, other studies reported that the Pro472Leu mutation, which is located in the D1–D2 linker, desensitized VCP/p97 without disrupting the binding sites of CB-5083 and NMS-873 but rather changed ATPase communication between the D1 and D2 domains, which is critical for their VCP/p97 inhibition capacity [183]. In colon cancer, the cytotoxicity of NMS-873 was suppressed by a VCP mutation located in the D2 domain (Ala530Thr) in NMS-873-resistant HTC116 cell lines. This mutation did not affect NMS-873 binding but increased VCP/p97 catalytic efficiency by altering ATP and ADP binding [184].

In 2020, Wang et al. selected CB-5083-resistant HCT116 colon cancer cell lines and analyzed the effect of a group of VCP/p97 inhibitors on these CB-5083-resistant cells [185]. These cells contained two VCP/p97 mutations (Asn660Lys and Thr688Ala) located in the D2 domain and were resistant to DBeQ, ML240 and ML241. On the other hand, the inhibitory potency of NMS-873 and UPCDC-30245 was unaffected by these mutations [185]. These authors also established a CB-5083-resistant cell line that harbored a double mutation in VCP (Asp649Ala/Thr688Ala) located in the D2 domain and showed that CB-5083, NMS-873, and UPCDC-30245 inhibited the proliferation of the parental HCT116 cell line, and NMS-873 and UPCDC-30245 were 30 times more potent at inhibiting this CB-5083-resistant double mutated cell line than CB-5083 [185].

## 6. Conclusions and Future Perspectives

VCP is an AAA+ ATPase that, together with many cofactors and adaptors, plays an important role in cellular homeostasis by regulating autophagy, mitochondrial-associated degradation, morphological alterations in nuclear and Golgi membranes, ERAD, endosomal trafficking and chromatin-associated degradation [8,9,10,11,12,13,14,15,16,17]. Recently, as described in the second section, increased VCP expression has been detected in various cancers and correlated with progression, prognosis, and metastatic potential. Notably, this protein was found to be secreted in previous studies. In fact, serum VCP levels were measured in patients with ovarian carcinoma, non-Hodgkin’s lymphoma and breast, colon, pancreatic, lung, and prostate cancer [61]. Therefore, it may represent a new biomarker for the dynamic monitoring of cancer progression and therapeutic responses. Future studies by our group and other researchers will surely focus on its evaluation in different biological fluids, such blood/sera, urine, and/or saliva, to verify its clinical utility and applicability.

Another interesting point pertains to the advances in the discovery of VCP/p97 inhibitors and the resistance mechanisms associated with VCP mutations, as discussed in the third and fourth sections. Although many inhibitors have been developed in recent years, only two phase I clinical trials on CB-5083 (NCT02243917 and NCT02223598) were initiated, but were terminated due to adverse effects on vision [144]. These data indicate that the design of new VCP/p97 inhibitors or derivatives of existing drugs with stronger inhibitory activity represents an interesting challenge. In this context, our group is performing virtual screening experiments against different binding pockets in VCP/p97, aimed at the repurposing of FDA (Food and Drug Administration)-approved drugs that can be used in patients with cancer stratified based on VCP/p97 expression levels.

## Figures and Tables

**Table 1:** Summary of the correlations between high VCP/p97 levels and the different features reported for various cancers.

Cancer Type	Features with which Higher VCP/p97 Levels Correlated	References
Colorectal cancer	greater invasion depth (T3–4), presence of venous invasion, higher tumor stage (III and IV), and higher recurrence rate	[61]
	CEA diagnostic marker	[62]
	carcinogenesis and metastasis development in a xenograft model	[63]
	USP11 levels influence colon cancer drug resistance	[64]
Pancreatic cancer	lymph node metastasis and disease-free and overall survival	[65]
	low levels of miR-198 and poor patient outcomes	[66]
	high levels of Ki-67 and a malignant prognosis	[65]
Liver cancer	shorter disease-free and overall survival	[67]
	low levels of miR-129-5p and HCC development and progression	[68]
	high levels of Neat1 and HCC diagnosis and treatment	[69]
	sorafenib response in HCC cells	[70]
	PTPRO levels	[71]
Gastric cancer	greater tumor size, presence of vascular and lymphatic invasion, lymph node metastasis, and shorter overall and disease free survival	[72]
	cell survival, degradation of cellular regulators, and gastric carcinogenesis	[73,74]
	low levels of CHOP and DR5	[75]
Esophageal cancer	higher frequencies of lymph node metastasis, deeper invasion, metastasis, and shorter disease free and overall survival	[76]
	shorter overall survival	[77]
Breast cancer	shorter overall survival	[78]
	poor outcomes of triple-negative patients receiving chemotherapy	[79,80]
	lower survival rates	[81]
	expression of the SOX2 protein	[82]
Prostate cancer	poor prognosis and increased metastatic potential	[83]
	high levels of IL-6	[84]
	maintenance of mitochondrial activity	[85]
Lung cancer	shorter disease-free and overall survival	[86]
	NSCLC development, progression and metastasis	[87]
	low levels of miR-129 and NSCLC development and progression	[88]
	increased levels of ER stress and EMT markers, chemoresistance and shorter patient survival	[89]
Bone cancer	higher metastatic potential (LM8)	[90,91]
	expression of the Aurora B protein	[92]
	low levels of miR-129-5p and osteosarcoma development and progression	[93]
	autophagy, anoikis resistance and osteosarcoma metastasis	[94]
	apoptotic response modulated by SAP	[95]
	cellular transformation and tumorigenesis	[96]
Head and neck cancer	tumor stages, lymph node metastasis and shorter overall survival	[97]
	development of carcinoma in situ lesions and invasivity	[98]
	better 5-year disease-free survival rates for HPV-negative patients	[99]
	clinical outcomes of chemo-radiotherapy	[100]
Thyroid cancer	disease recurrence in patients with follicular thyroid carcinoma	[101]
	BPA exposure	[102]
	shorter disease-free survival and an increased risk of recurrence in patients with papillary thyroid cancer subjected to ablative radioiodine treatment	[103]
Hematological cancer	tumor grade, stage, histological subtype, recurrence and shorter overall and disease-free survival of patients with B-cell lymphoma	[104]
	multiple myeloma development and progression	[105]
	poor prednisone responders in pediatric patients with acute lymphoblastic leukemia	[106]
	exosome generation and secretion in Jurkat tumor cells	[107]
Melanoma	advanced radiotherapy	[108]
	immune escape	[109]
Glioblastoma	radiosensitivity of glioblastoma cells, and survival time of xenografted mice with radiation treatment	[110,111]
	HDAC6 levels and temozolomide resistance therapy	[112]
Ovarian cancer	chemotherapy response in patients receiving the platinum-taxane combination	[113]
Testicular cancer	development of different types of human testicular tumors	[114]
Bladder cancer	shorter survival following bladder removal by cystectomy	[115]
Squamous cell carcinoma	development of squamous cell carcinoma	[116]

**Table 2:** Analysis of somatic mutations in VCP performed using the COSMIC database [181].

	Number	Location
Deletion-frameshift	2	both in the D2 domain
Deletion-in-frame	1	D1 domain
Insertion-frameshift	2	1 in D2 and 1 in the C-terminal domain
Substitution-coding silent	65	14 in the N-terminal domain, 1 in the N-D1 linker, 19 in D1, 4 in the D1-D2 linker, 24 in D2 and 3 in the C-terminal domain
Substitution-missense	185	40 in the N-terminal domain, 7 in the N-D1 linker, 61 in D1, 3 in the D1-D2 linker and 74 in the D2 domain
Substitution-nonsense	15	6 in the N-terminal domain, 3 in D1 and 6 in the D2 domain
